# The Transmittance Modulation of ZnO/Cu/ZnO Transparent Conductive Electrodes Prepared on Glass Substrates

**DOI:** 10.3390/ma13183916

**Published:** 2020-09-04

**Authors:** Dooho Choi

**Affiliations:** School of Advanced Materials Engineering, Dong-Eui University, 176 Eomgwangro, Busan 47340, Korea; dhchoi@deu.ac.kr

**Keywords:** transparent electrodes, sputtering, copper, ZnO, transmittance

## Abstract

With the explosive development of optoelectronic devices, the need for high-performance transparent conductive (TCE) electrodes for optoelectronic devices has been increasing accordingly. The two major TCE requirements are (1) visible light average transmittance higher than 80% and (2) sheet resistance lower than 10 Ω/sq. In this study, we investigated the critical role of the top and bottom ZnO thicknesses for the ZnO/Cu/ZnO electrodes prepared on glass substrates. It was shown that the required Cu thickness to meet the conductivity requirement is 8 nm, which was fixed and then the thicknesses of the top and ZnO layers were independently varied to experimentally determine the optimized conditions for optical transparency. The thicknesses of the top and bottom ZnO layers were both found to significantly affect the peak transmittance as well as the average visible light transmittance. The ZnO/Cu/ZnO electrode exhibits peak and average transmittance of 95.4% and 87.4%, excluding the transmittance of glass substrates, along with a sheet resistance of 9.7 Ω/sq, with a corresponding Haacke’s figure of merit ($\varphi_{H}=\frac{T_{ave}^{10}}{R_{s}}$) of 0.064, which exceeds the reported value for the ZnO/Cu/ZnO electrodes, manifesting the need of experimental optimization in this study.

## 1. Introduction

With the explosive development of optoelectronic devices such as light emitting diodes and solar cells, the need of highly transparent and conductive transparent conductive electrodes (TCE) is accordingly increasing [1,2,3] The general TCE requirements include visible light transmittance of higher than 80% and the sheet resistance in the of lower than 10 Ω/sq [4,5] The conventional TCE material satisfying these requirements is indium tin oxide (ITO), but such properties can be obtained only via an annealing process at temperatures higher than 250 °C, [6] which significantly increases the fabrication cost and time in addition to the inherent incompatibility with heat sensitive flexible polymer substrates such as polyethylene terephthalate (PET) films. In order to overcome the limitations of ITO, various novel materials and nanostructures, such as carbon nanotudes [7,8] graphene [9,10] metallic nanowires [11,12], and poly(3,4-ethylenedioxythiophene):poly(styrenesulfonate) [13], have been proposed. Recently, oxide/metal/oxide (OMO) type of TCEs has gained much attention by noting the low resistivity of metals and high optical transparency in the form of ultrathin layers [14,15,16].

The transmittance and sheet resistance of the OMO electrodes have a trade-off thickness dependence, and it is known that the maximum transmittance can be achieved when the metal layers becomes nearly continuous and further metal layer thickening reduces the transmittance following the Beer’s law, *I_T_* = *I_0_e^−^^βd^* where *I_0_*, *I_T_*, *β* and *d* are incident light intensity, transmitted light intensity, absorption coefficient and sample thickness, respectively. Therefore, one of the primary goals for the OMO electrode research so far has been methodology development to suppress the Volmer–Weber 3D thin film growth mode so that the continuous layer formation occurs at metal layer thicknesses as thin as possible. These approaches utilized various novel growth techniques including surface modification of polymer substrates before metal layer deposition [14], trace-level gas (O_2_ and N_2_) addition during metal sputtering [15] and impurity cosputtering [16]. While Ag is the primary material of choice for the OMO electrodes due to its low resistivitiy (1.6 μΩ-cm) and high visible-light transmittance in the form of an ultrathin (~10 nm) layer, TCEs are one of the most expensive components in optoelectronic devices and thus it is desirable to replace Ag with low cost electrode materials [17] Cu has been recently considered as a promising alternative due to its low resistivity (1.7 μΩ-cm) that is comparable to that of Ag and its low material cost that is only ~1% relative to that of Ag [18]. While the TCE conductance is mostly determined by the metal layer, the top and bottom oxides serve as anti-oxidation and anti-reflection coatings and a variety of oxide layers such as MoO_3_, Bi_2_O_3_, and ZnO have been studied to fulfill the purposes [19]. However, systematic studies on the experimental verification of the thickness dependence of the top and bottom oxides for the ZnO/Cu/ZnO electrodes have not been conducted, although the transmittance modulation of the TCE structure was predicted by optical simulation [20]. This study reports the dramatic modulation of the TCE transmittance with the independent variations of the top and bottom oxide layers, which results in the maximum and average transmittance for the optimized ZnO (40 nm)/Cu (8 nm)/ZnO TCE (40 nm) being 95.4% and 87.4%, excluding the transmittance of glass substrates, along with the sheet resistance of 9.7 Ω/sq. The corresponding Haacke figure of metrit (*Φ_Haacke_* = *T*^10^/*R_s_*) is 0.064, which is higher than reported values for Cu-based OMO electrodes.

## 2. Experiment

The multi-layers of ZnO/Cu/ZnO electrodes were deposited from 3-inch targets of Cu (99.999 wt%) and ZnO (99.999 wt%) onto glass substrates (Corning E2000). The sputtering chamber was evacuated to a base pressure of 3 × 10^−7^ torr, and then elevated to and maintained at 6 × 10^−3^ torr with the inflow of 99.9999% Ar gas. The Cu and ZnO targets were then sputtered at a direct current (DC) power of 50 W and a radio frequency power of 50 W, respectively. The substrates were rotated at a speed of 15 revolutions per minute in order to promote spatial thickness uniformity. The substrates were not intentionally heated or cooled during deposition. Deposition rates were measured by Surface Profiler (D-100, KLA, Milpitas, CA, USA) using films of ZnO and Ag that are thicker than 200 nm, which shows a nearly linear relation between the layer thickness and deposition time with the coefficient of determination (R^2^) values higher than 0.99. The deposition rates were found to be 2.6 Å/s and 0.56 Å /s for Cu and ZnO, respectively, which were then used to determine the thicknesses of the layers. Even for the non-continuous Cu layers, the thicknesses given are based on the deposition rate for the thick, continuous films. The Cu layer thickness was fixed at 8 nm, whereas the top and bottom ZnO layer thicknesses were independently varied in the range of 0–70 nm.

Sheet resistance and the corresponding film resistivity of the Cu layers were measured using the four point probing method. Transmittance of visible light (wavelengths in the range of 400–800 nm) was measured using spectrophotometry (CARY-100, Agilent, Santa Clara, CA, USA). The reproducibility of the electrode properties were verified by preparing 3 samples having the identical structure of ZnO (30 nm)/Cu (8 nm)/ZnO (30 nm), which showed deviations from the average values only by 1.1% and 0.2% for the sheet resistance and visible light transmittance, respectively. The surface morphologies of the Cu and ZnO layers were observed using ultrahigh-resolution field-emission scanning electron microscopy (SEM) (S-5500, Hitachi, Tokyo, Japan) operated at 5 kV. The cross-sectional ZnO/Cu/ZnO electrode structure was observed with transmission electron microscopy (TEM; TECNAI F20, FEI, Hillsboro, OR, USA) operated at 200 kV. For the Cu samples, the top ZnO layers were not deposited to allow for the direct observation of the Cu layer morphology. 100 nm-thick Ag electrodes were formed along the opposite far sides of the electrodes for Joule heating test, where external DC bias was applied (EPS-3305, EZT, Busan, South Korea) and the electrode temperature was measured by infrared (IR) camera (PTI120, Fluke, Science Park, Amsterdam, The Netherlands).

## 3. Results and Discussion

**Figure 1:**
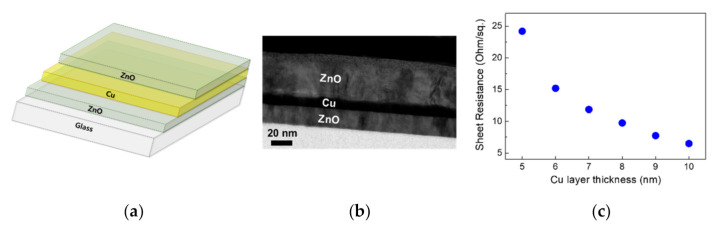

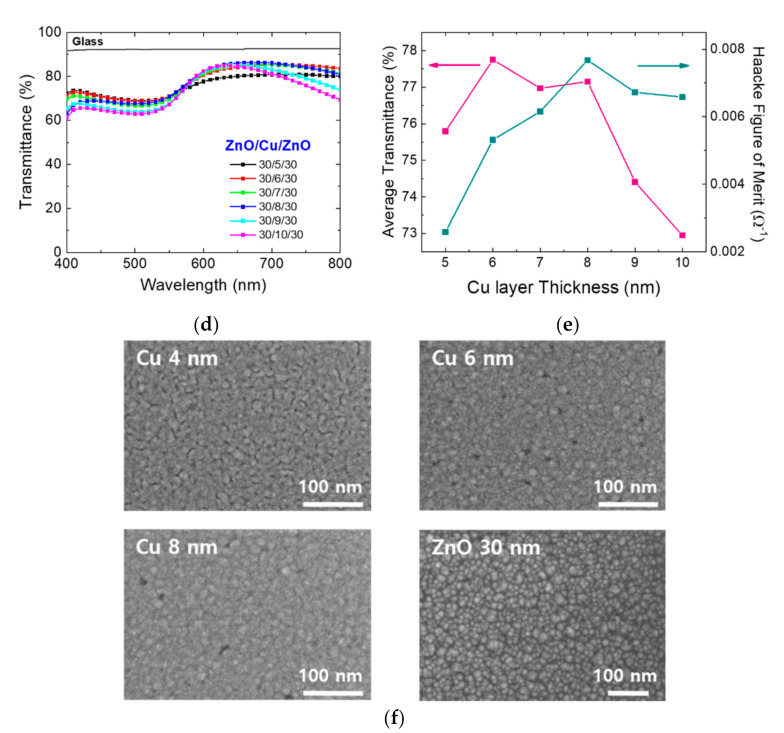
(**a**) Schematic structure of ZnO/Cu/ZnO electrodes. (**b**) A cross-sectional TEM micrograph for ZnO (20 nm)/Cu (8 nm)/ ZnO (40 nm). (**c**) Sheet resistance of the electrodes as a function of Cu layer thickness. The top and bottom ZnO layers are both fixed at 30 nm. (**d**) The transmittance of the ZnO/Cu/ZnO electrodes as a function of Cu layer thickness with the top and bottom ZnO thicknesses both kept at 30 nm. The air baseline was taken and the transmittance of bare glass is also given as reference. The air baseline was taken. (**e**) The average visible light transmittance and the Haacke figure of merit values (evaluated with the average visible light transmittance) are plotted together. The highest Haacke figure of merit is obtained with the Cu layer thickness of 8 nm. (**f**) SEM micrographs showing the morphologies of Cu layers having thicknesses of 4, 6 and 8 nm, respectively, for which the top ZnO layer was not deposited for direct observation of the Cu layers. An SEM image of the top ZnO layer for the ZnO (30 nm)/Cu (8 nm)/ZnO (30 nm) electrode was also shown.

A schematic structure of the ZnO/Cu/ZnO electrode prepared onto a glass substrate is illustrated in Figure 1a along with a TEM micrograph showing each layer of the electrode in Figure 1b. The sheet resistance and resistivity values for the OMO electrodes as a function of Cu layer thickness are given in Figure 1c. As the sheet resistance of the ZnO layers exceeds the limit (2 MΩ/sq.) of the four point probe system, the conductivity of the OMO electrodes can be considered to be determined by the Cu layers, whose sheet resistance values were found to be independent of the bottom ZnO thickness. In Figure 1d, the evolution of Cu morphology with increasing layer thickness from 4 to 8 nm shows that the nearly continuous Cu layer is formed at the thickness of ~8 nm, above which the sheet resistance (9.7 Ω/sq.) was found to meet the typical TCE conductivity requirement (<10 Ω/sq.). Figure 1d presents visible light transmittance measured for Cu layers having thicknesses of 5–10 nm with top and bottom ZnO layer thicknesses fixed at 30 nm, and the average transmittance values in addition to the Haacke figure of merit are plotted in Figure 1e. The highest figure of merit coincides with the Cu layer thickness of 8 nm. For the remainder of this study, the Cu layer thickness for the ZnO/Cu/ZnO electrodes was fixed at 8 nm, and the impact of thickness variations for the top and bottom ZnO layers for optimized visible light transmittance will be investigated, by independently varying the top and bottom ZnO layer thickness. In Figure 1f, SEM micrographs show the morphological evolution of the Cu layers with increasing thickness from 4 to 8 nm, respectively. A nearly continuous Cu layer was shown to be formed at the thickness of 8 nm. An SEM image of the top ZnO layer for the ZnO (30 nm)/Cu (8 nm)/ZnO (30 nm) electrode is also shown, wherein no pinholes were observed.

**Figure 2:**
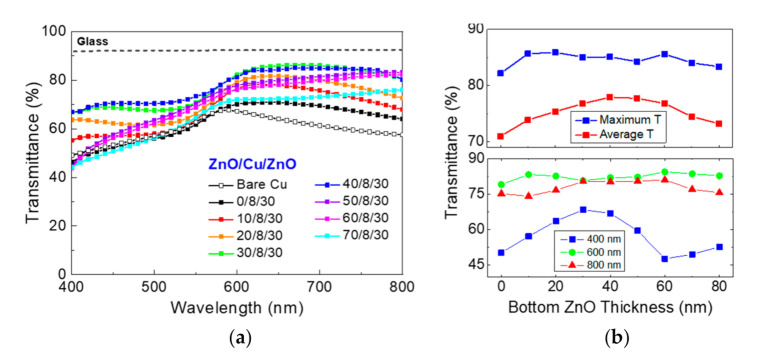
(**a**) Optical transmittance for the ZnO/Cu/ZnO electrodes as a function of incident beam wavelength. The thickness of top ZnO and Cu layers are fixed at 30 nm and 8 nm, respectively, while the bottom ZnO layer thicknesses were varied in the range of 0–70 nm. The transmittance of a bare Cu layer, i.e., having no top and bottom ZnO layers, is also included for comparison. The air baseline was taken and the transmittance of bare glass is also given as reference. (**b**) (upper panel) Maximum and average transmittance for the ZnO/Cu/ZnO electrodes are given as a function of bottom ZnO layer thickness. (lower panel) The transmittance evaluated at the wavelengths of 400, 600 and 800 nm are given, respectively, as a function of the bottom ZnO layer thickness.

Figure 2a shows the visible light transmittance for the ZnO/Cu/ZnO electrodes having the bottom ZnO layer thicknesses in the range of 0–80 nm, whereas the top ZnO layer thickness was fixed at 30 nm. The bare 8 nm-thick Cu layer, i.e., with no top and bottom ZnO layers, shows significantly lower visible-light transmittance, clearly suggesting the need of sandwich oxide layers. For the assessment of the impact of thickness variation for the top ZnO, the maximum transmittance and average visible light transmittance in addition to the transmittance evaluated at 400 nm (blue), 600 nm (green) and 800 nm (red) are presented in Figure 2b. While the maximum transmittance does not have a particular trend, the average transmittance exhibits the peak transmittance at the bottom ZnO thickness of 40 nm, primarily due to the rather significant thickness dependence of the transmission associated with the relatively short wavelength, as evidenced for the transmittance of photons having the wavelength of 400 nm in the lower panel in Figure 2b. Based on the overall transmittance and spectral uniformity, the optimum top ZnO thickness for ZnO (30 nm)/Cu (8 nm)/ZnO (0–80 nm) was determined to be 40 nm.

**Figure 3:**
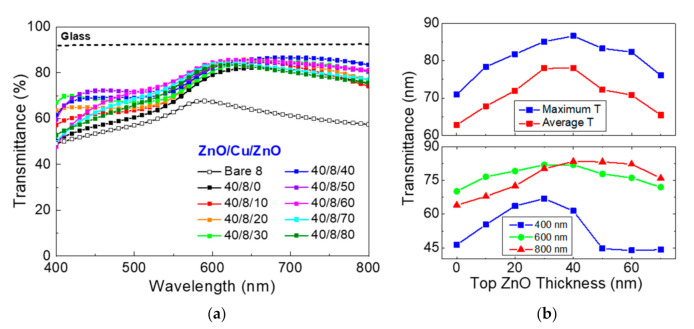
(**a**) Optical transmittance for the ZnO/Cu/ZnO electrodes as a function of incident beam wavelength. Top ZnO layer thicknesses are varied from 0 to 80 nm, with the bottom and Cu layer thickness fixed at 40 nm. The transmittance of bare Cu layer (8 nm) is included for comparison. The air baseline was taken and the transmittance of bare glass is also given as reference. (**b**) (upper panel) Maximum and average transmittance for the ZnO/Cu/ZnO electrodes are given as a function of top ZnO layer thickness. (lower panel) The transmittance evaluated at the wavelengths of 400, 600 and 800 nm are given, respectively, as a function of the top ZnO thickness.

The impact of the top ZnO layer thickness was also evaluated by varying the thickness in the range of 0–80 nm, with the bottom ZnO thickness fixed at 40 nm. The modulated transmittance spectra associated with the thickness variation is given in Figure 3a, which again clearly suggests the need of thickness optimization for the bottom ZnO layer. In Figure 3b, the maximum and average transmittance as well as the transmittance evaluated at the wavelengths of 400, 600, and 800 nm are presented. In contrast to the trend of the bottom layer thickness, the maximum and average transmittance in Figure 3b both exhibit clear thickness dependence with the peaks located at ~40 nm. The wavelengths associated with the maximum transmittance (40 nm) and the highest average transmittance (30 nm) are found to be slightly different, as the transmittance for the short (400 nm), middle (600 nm), and long (800 nm) wavelengths have peaks at different top ZnO layer thickness.

**Figure 4:**
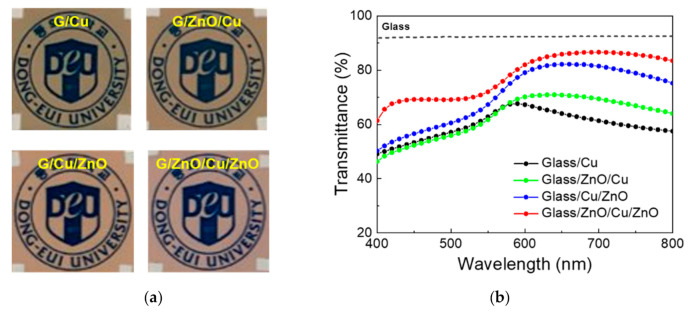
(**a**) Photographs of logos that are placed below the electrode structures of (1) glass/Cu (8 nm), (2) glass/ZnO (30 nm)/Cu (8 nm), (3) glass/Cu (8 nm)/ZnO (40 nm) and (4) glass/ZnO (40 nm)/Cu (8 nm)/ZnO (40 nm), respectively. (**b**) Transmission comparison for the four electrode structures. The air baseline was taken and the transmittance of bare glass is also given as reference.

The critical roles for the top and bottom ZnO oxides become immediately clear by comparing the photographs where four types of electrodes, i.e., Cu, ZnO/Cu, Cu/ZnO and ZnO/Cu/ZnO are placed above the same logos in Figure 4a. It is noteworthy that the presence of top and bottom ZnO layers noticeably strengthen the bluish color, in agreement with the transmission comparison in Figure 4b, where the transmission of low-wavelength (400–550 nm) photons significantly increased with the insertion of both the top and bottom ZnO layers.

Table 1 lists the thicknesses of the top/bottom ZnO layers, with the resultant maximum transmittance values along with the corresponding peak wavelengths and the average visible light transmittance over the wavelengths of 400–800 nm. The electrode transmittance values are presented with the transmission loss of the glass substrates excluded. The modulation of the electrode transmittance is resulted by the complex interference of the reflected light at various interfaces present in the electrodes, i.e., air/top ZnO, top ZnO/Cu, Cu/bottom ZnO, bottom ZnO/glass, and bottom ZnO/air. While reflectivity at an interface between two media having refractive indices of *n*_1_ and *n*_2_ can be computed using the relation of $\frac{{\left(n_{1}-n_{2}\right)}^{2}}{{\left(n_{1}+n_{2}\right)}^{2}}$, it is well known that the optical properties of thin films can be significantly different from the theoretical prediction due to various factors including interface roughness, film texture, grain boundaries, dislocations, point defects and impurities [21]. Therefore, it is essential to experimentally conduct thickness optimization for OMO electrodes composed of multiple thin layers prepared by a particular deposition method. The resultant achievement of 95.4% of maximum transmittance along with sheet resistance of 9.7 Ω/sq. should be highlighted. The TCE Haacke figure of merit for the optimized TCE is computed to be 0.064, which is higher than reported values [17], manifesting the need of thickness optimization in this study. Further research efforts, such as the introduction of novel oxide layers and methodology development to promote the wettability of metallic layers, are expected to further improve the OMO structure TCE properties.

**Figure 5:**
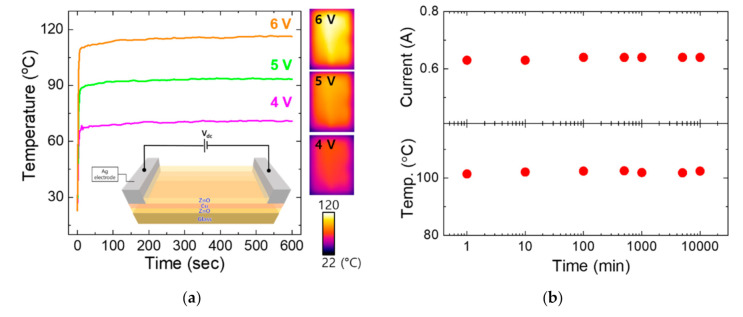
(**a**) Temperature profiles for the ZnO/Cu/ZnO electrodes in response to given external DC voltages, and the IR images corresponding to the applied voltages are also given. The schematic electrical setup was given as an inset. (**b**) Current (upper panel) and temperature (lower panel) as a function of time while the electrode temperature was maintained at ~100 °C.

The TCE electrodes having excellent transparency and conductivity can also be used as high-performance transparent heaters for various applications, either as an integrated component for optoelectronic devices e.g., for the removal of snow on solar panels [22] or as a stand-alone defrosting device, e.g., for vehicles and windows [23,24]. Figure 5a shows the heater characteristics for the ZnO/Cu/ZnO electrodes having 6 nm-, 8 nm-, and 10 nm-thick Cu layers. It was shown that the heater temperatures and electrical currents at given voltages are inversely proportional to the TCE sheet resistance, as is expected by the Joule heating mechanism. To evaluate the structural integrity over time, an endurance test was performed for the electrode, for which a constant DC bias is applied for up to 10,000 min with the heater temperature maintained at ~100 °C in air. No changes have been observed with the heater temperature and electrical current during the test, which clearly indicates that oxidation of Cu layers is minimal even at the temperature significantly higher than room temperature owing to the dense ZnO layer efficiently serving as an anti-oxidation layer. The superb TCE characteristics optimized in this study combined with the excellent heating capability are expected to be highly appreciated for various optoelectronic applications.

## 4. Conclusions

A significant transmittance modulation for ZnO/Cu/ZnO transparent conductive electrodes associated with the alteration of ZnO layer thicknesses was systematically investigated. With the Cu layer thickness fixed at 8 nm, at which the sheet resistance was found to be lower than the typical requirement of 10 Ω/sq., the thicknesses of the top and bottom ZnO layers were independently varied to arrive at the optimized visible light transmittance. The thicknesses of the top and bottom ZnO layers were both found to significantly affect the peak transmittance as well as average visible light transmittance. With this experimental verification the highest transmittance of 95.4% and average transmittance of 87.4 (excluding the transmittance of glass substrates) at the thickness of 40 nm for both of the top and bottom ZnO layers were achieved, with the corresponding Haacke figure of merit of 0.064. This is higher than the reported value for the identical structure, manifesting the need of thickness optimization adopted in this approach.

## Figures and Tables

**Table 1:** Sample number, layer thicknesses of the bottom ZnO, Cu and top ZnO. The maximum transmittance values with the corresponding wavelengths and the average visible light transmittance values are presented, for which the transmission loss for the glass substrates was excluded.

Sample No.	Layer Thickness (nm)			Visible Light Transmittance	
	Bottom ZnO	Cu	Top ZnO	Max (Wavelength)	Average (λ: 400–800 nm) (%)
1	0	8	0	76.5 (590 nm)	68.8
2	0		30	91.1 (650 nm)	80.5
3	10			94.5 (640 nm)	83.2
4	20			94.7 (650 nm)	84.6
5	30			93.9 (680 nm)	85.3
6	40			94.0 (670 nm)	87.1
7	50			93.1 (670 nm)	87.1
8	60			94.4 (640 nm)	86.5
9	70			92.9 (620 nm)	84.0
10	80			92.3 (620 nm)	82.7
11	40		0	79.7 (630 nm)	72.1
12			10	87.1 (630 nm)	77.0
13			20	90.6 (650 nm)	81.0
14			30	94.0 (670nm)	87.1
15			40	95.4 (700 nm)	87.4
16			50	92.0 (790 nm)	82.1
17			60	91.1 (790 nm)	80.6
18			70	84.8 (800 nm)	75.1
